# Evaluation of Adjuvant Administration of a Prophylactic HPV Vaccine in HPV-Positive Infertile Men: A Systematic Review

**DOI:** 10.3390/vaccines14090778

**Published:** 2026-09-06

**Authors:** Ana Banko, Ivana Lazarevic, Danijela Miljanovic, Marko Jankovic, Jovana Cupic, Andja Cirkovic

**Affiliations:** 1 Institute of Microbiology and Immunology, Faculty of Medicine, University of Belgrade, 11000 Belgrade, Serbia; ivana.lazarevic@med.bg.ac.rs (I.L.); danijela.karalic@med.bg.ac.rs (D.M.); marko.jankovic@med.bg.ac.rs (M.J.); 2 Clinic for Gynecology and Obstetrics, University Clinical Center of Serbia, 11000 Belgrade, Serbia; 3 Institute for Medical Statistics and Informatics, Faculty of Medicine, University of Belgrade, 11000 Belgrade, Serbia; andja.cirkovic@med.bg.ac.rs

**Keywords:** human papillomavirus (HPV), semen infection, HPV vaccination, male infertility, sperm quality

## Abstract

**Background:** Accumulating data suggest that human papillomavirus (HPV) infection may be associated with male infertility by impairing sperm quality. An induced anti-HPV humoral immune response could mediate the effective clearance of HPV infection. This systematic review aimed to assess reported differences in semen quality parameters and HPV presence in men with persistent HPV infection after adjuvant administration of a prophylactic HPV vaccine. **Methods:** Possibly eligible articles were retrieved from three electronic databases (PubMed, Scopus, and Web of Science) since 26 June 2026, with three ultimately included in both qualitative and quantitative analysis. **Results:** The estimated HPV DNA negativity was 74% (95% CI 59–86) and 98% (95% CI 78–100) at the end of vaccination and 6 months after complete vaccination. **Conclusions:** This study identified limited evidence suggesting a potential benefit of HPV vaccination in infertile HPV-positive men, particularly in terms of shift toward HPV negativity. Given the small number of available studies and their methodological limitations, further large, well-designed prospective trials are required to determine whether adjuvant administration of a prophylactic HPV vaccine provides clinically relevant benefits in this population.

## 1. Introduction

Human papillomavirus (HPV) is considered the most prevalent sexually transmitted virus worldwide, with nearly every individual likely to be infected at least once during their lifetime [1,2]. The virus is primarily transmitted horizontally, mainly through a sexual act—sexual penetration or intimate genital contact—and it can be found in the uterus, cervix, and external genitalia of both sexes as well as the anogenital, oral, and cutaneous regions [3,4]. Due to the asymptomatic course of infection, it commonly goes unnoticed, and in 90% of cases it is cleared by the immune system within 24 months of primary exposure [5]. However, if persistence is established, HPV-induced lesions, due to viral oncogenic potential, might progress to carcinoma. In particular, 95% of cervical cancers, the fourth most common cancer in women globally, are related to HPV [2].

Although women have been the primary focus of studies addressing HPV-related diseases, growing attention is being directed toward the role of HPV infection in men. The estimated overall prevalence for genital HPV infection in men is 31%, with HPV-16 being the most common high-risk HPV type (HR-HPV) [6]. In fact, infection with HR-HPV is an established etiological factor for nearly all anal cancers, a substantial proportion of penile cancers, and a large subset of oropharyngeal squamous cell carcinomas [7]. Due to immune system dysfunction, HPV infection poses unique challenges for HIV-positive individuals [8]. Compared to people without HIV, those who are HIV positive had a higher prevalence and incidence of HPV infection including HPV-16 and HPV-related diseases, with the prevalence of anal HPV infection ranging between 44% and 83%, higher in men, and highest in HIV-positive MSM [9,10]. Additionally, HPV in males causes cutaneous warts and respiratory papillomatosis, and also invades accessory glands, including the seminal vesicles, prostate gland, and bulbourethral glands [11,12].

Accumulating data increasingly suggest that HPV infection may be associated with infertility in both males and females, affecting human reproduction [13,14,15]. In semen, particularly, the prevalence of HPV DNA is highly variable, from 0 to 46.2%, yielding an average of 17% [15]. As a widely accepted origin of seminal HPV, the penile shaft, coronal sulcus/glans penis, and the scrotum collectively account for more than 95% of all detected genital HPV infections among asymptomatic heterosexual men [16]. The possible mechanisms behind the role of HPV presence in semen and its link to infertility are still not fully understood and are currently being investigated. However, studies have shown that the prevalence of HPV in seminal fluid from infertile men is 3 to 4 times higher than in fertile controls [17,18].

Based on available literature evidence, it has been proposed that HPV infection directly impairs sperm quality, which is considered one of the main risk factors determining the prediction of conception success [17,19,20]. Researchers described reduced sperm progressive motility as the most significant consequence, followed by sperm DNA integrity; sperm count, concentration, viability, and morphology; semen volume; and elevated levels of anti-sperm antibodies (ASA) [13,14,15].

As HPV DNA can be detected in various fractions of semen, including seminal plasma, immune cells, semen cells, and somatic cells [21], many hypotheses have been proposed to explain the mechanism between HPV and fertility impairment. Some authors have proposed that this may be related to the presence of HPV in the equatorial region of the sperm head [22]. Therefore, the sperm could serve as a carrier of the virus all the way to the placenta and embryonal cells, compromising attachment of the trophoblasts and pregnancy [22,23]. In addition, HPV could directly damage host cell DNA, leading to genomic instability or apoptosis [13,23]. Finally, the anti-HPV immune response produced during natural infection could mediate clearance of HPV-labeled structures, from spermatozoa and oocytes to HPV-infected embryos [23].

It has been suggested that seminal fluid represents a sample of high diagnostic value to evaluate the presence of HPV in asymptomatic individuals [24]. In addition, the level of serum anti-HPV antibodies (Abs) was identified as the most valuable prognostic factor related to HPV DNA clearance [25]. Because prophylactic anti-HPV vaccination relies on the induction of a humoral immune response and is highly effective in preventing HPV-associated diseases in both women and men [26,27,28], a new hypothesis has emerged regarding the therapeutic HPV vaccine’s effectiveness. Based on the available evidence, some studies suggest that adjuvant administration of a prophylactic HPV vaccine may constitute active immunotherapy, which is associated with an increased rate of natural pregnancies and live births [14]. Therefore, this systematic review aimed to assess reported differences in semen quality parameters and HPV presence in HPV-positive men after HPV vaccination.

## 2. Materials and Methods

The actual research, registered as a systematic review (PROSPERO registration number: CRD420251272924), was performed in accordance with the PRISMA protocol (Reporting Items for Systematic Reviews and Meta-Analyses) [29].

### 2.1. Search Strategy and Study Selection

All studies that evaluated the effect of the anti-HPV vaccine on any semen parameter (sperm concentration, total sperm count, progressive motility, normal morphology, viability, IgG or IgM antibodies, and HPV in semen) among previously HPV-positive male respondents were included in this systematic review. Reasons for exclusion were: (1) publication in language other than English, (2) non-original articles (narrative reviews, systematic reviews, meta-analysis, case reports, case series, editorials, comments, correspondences, books, short, abstracts, etc.), (3) population other than humans (animals, cell lines), (4) vaccine other than anti-HPV vaccine, and (5) semen characteristics of interest were not evaluated at all.

Two experienced researchers in the field of conducting systematic reviews and meta-analyses (AC, AB) developed and performed the search strategy within three major electronic databases: PubMed, Web of Science (WoS), and SCOPUS on 26th June 2026. Search strategies were constructed according to the PICOS research framework: Population—male human HPV-positive respondents of all ages, Intervention—anti-HPV vaccine, Control—placebo or no anti-HPV vaccine, Outcome—sperm concentration, total sperm count, progressive motility, normal morphology, viability, IgG or IgM antibodies, and HPV DNA in semen, Study design—clinical trials, prospective or retrospective cohorts, nested case–control studies in cohorts, case–controls, and cross-sectional studies. They are presented in detail in Table 1.

Reference lists of all review articles identified through electronic retrieval were manually searched to identify all potentially relevant publications for inclusion.

### 2.2. Article Screening and Selection

Two independent reviewers (DM, IL) checked the eligibility of collected publications through the title and abstract reading step. Studies were included in the full-text screening if either reviewer identified the study as being potentially eligible or if the abstract and title did not include sufficient information for exclusion. The same reviewers independently performed full-text readings to select articles for inclusion according to previously defined inclusion and exclusion criteria. Disagreements were resolved by consensus (DM, IL) or arbitration (AB).

### 2.3. Data Abstraction and Quality Assessment

Two reviewers (DM, IL) independently abstracted the following data: (1) study characteristics (author, year of publication, country of research, study design), (2) population characteristics (number of cases, age, detailed characteristics, inclusion and exclusion criteria), (3) vaccine characteristics (type and number of doses of the applied vaccine), (4) outcome characteristics (parameter, sample for the analysis, method applied), and (5) follow-up characteristics (time points of the outcome assessment). All data were collected according to a previously designed extraction protocol. Study quality was assessed by two reviewers independently. The adapted version of the Newcastle–Ottawa tool (NOS) was used for observational studies [30], while the Jadad scale was used for clinical trials [31].

### 2.4. Statistical Analysis

Categorical data were presented as absolute and relative numbers in percentages, while numerical data were reported as in the original article.

The major goal of this study was to evaluate the change in semen parameters during the follow-up period after vaccination in previously HPV-positive men. Two outcomes could be evaluated: the prevalence of HPV DNA negativity from baseline to 6 months and from baseline to 12 months after recruitment in vaccinated male respondents, and the difference in frequency of HPV DNA positivity between cases (vaccinated HPV-positive male participants) and controls (unvaccinated HPV-positive male participants) at 6 and 12 months after recruitment, while differences in semen parameters neither over time within vaccinated respondents nor between vaccinated and unvaccinated male respondents could be performed. Within the second outcome evaluation, sensitivity analyses were performed by considering, separately, the seronegative and seroconverted controls from Foresta et al. [32]. Results are presented as forest plots showing the proportion of negativity or odds ratio (OR) (box), 95% confidence interval (lines), and study weight (size of the box) for each of the included studies, together with the pooled effect measure (diamond). The pooled effect measure depended on the type of outcome and comparison: in the first meta-analysis, it was the proportion of HPV DNA negativity, whereas in the second, it was an odds ratio (OR) for HPV DNA positivity. Heterogeneity was assessed by the I^2^ statistic. Considering the clinical and methodological heterogeneity among the included studies and the small number of studies available, a random-effect model was used for the meta-analysis. The choice of model was not based solely on a statistical test of heterogeneity, as the estimation of between-study heterogeneity is imprecise when only a small number of studies are available. The assessment of the overall risk of bias and methodological quality of the included observational studies was performed by the Newcastle–Ottawa Scale (NOS). Study quality: According to the Newcastle–Ottawa Scale (NOS) criteria, studies were categorized as good quality if they received 3 or 4 stars in the Selection domain, 1 or 2 stars in the Comparability domain, and 2 or 3 stars in the Outcome/Exposure domain, or had a total score of ≥7 stars. Fair quality was defined as 2 stars in the Selection domain, 1 or 2 stars in the Comparability domain, and 2 or 3 stars in the Outcome/Exposure domain, or a total score of 5–6 stars. Poor quality was defined as 0 or 1 star in the Selection domain, 0 stars in the Comparability domain, or 0 or 1 star in the Outcome/Exposure domain, or a total score of ≤4 stars. Assessment of publication bias using funnel plot asymmetry and formal statistical tests was not performed, in accordance with the recommendations of Sterne et al. [33], which generally recommend assessing funnel plot asymmetry only when at least 10 studies are available. Since only three studies were included in the meta-analysis, these methods would have insufficient statistical power and may yield unreliable results. The minimum recommended number of studies required to perform a meta-analysis is two to three [34], although the validity of the results is limited by the study’s small power, whilst the validity of the results depends on study heterogeneity, quality, and number of respondents. A *p*-value < 0.05 was considered statistically significant. Analyses were performed using R version 4.6.1 (R Foundation for Statistical Computing, Vienna, Austria) in RStudio version 2026.06.0 (Build 242).

## 3. Results

### 3.1. Feasibility Assessment of Systematic Review and Meta-Analysis

The current topic could be beneficial not only for doctors and scientists but even more for patients, which is why it was necessary to conduct a feasibility analysis of systematic review and meta-analysis in order to provide a rationale for conducting this study (Table 2).

### 3.2. Systematic Review

**Figure 1:**
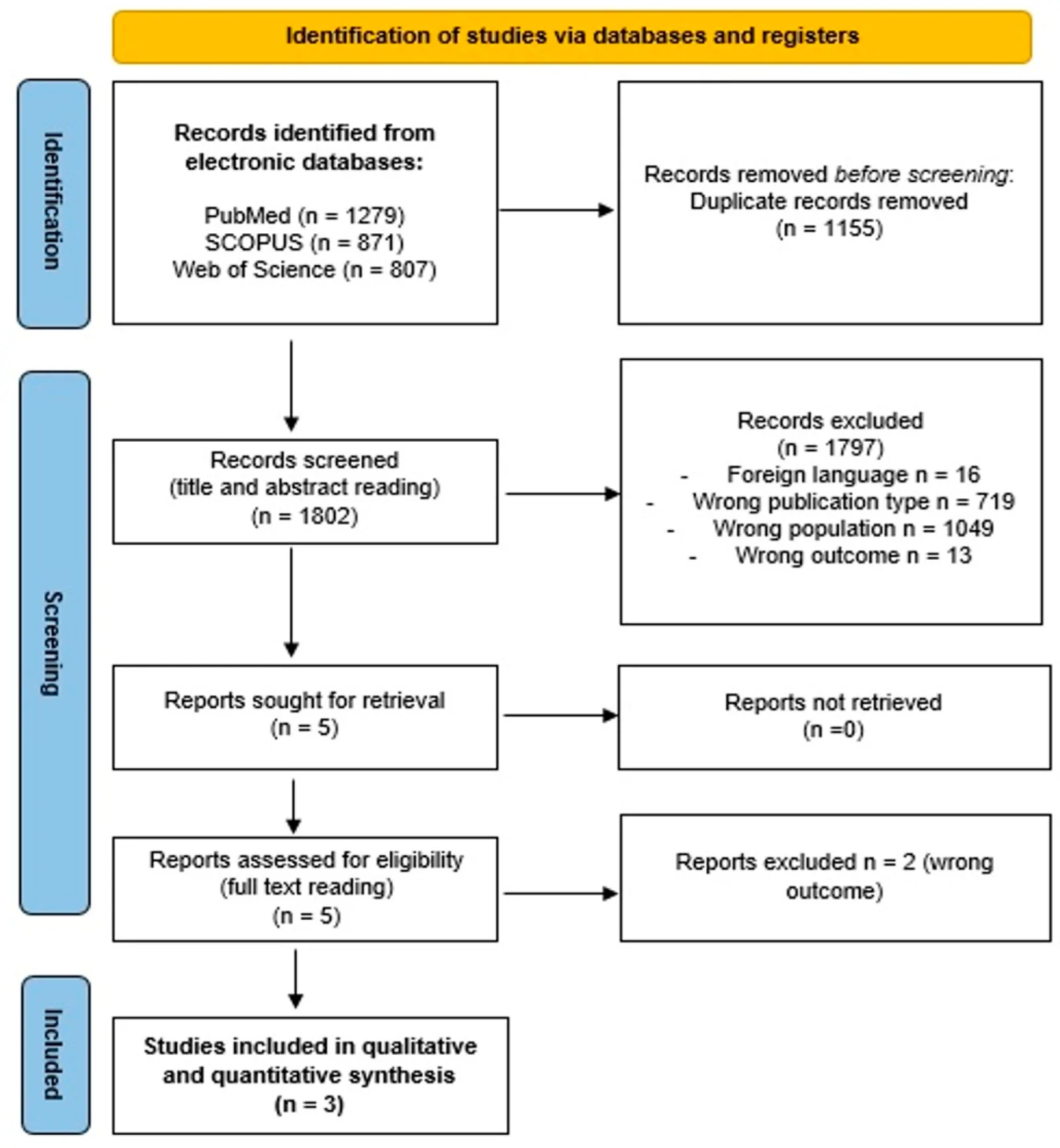
Flow diagram.

A total of 2957 potentially eligible articles were collected from three electronic databases. After 1155 duplicates were removed, 1802 titles and abstracts were screened for inclusion. After the exclusion of 1797 articles (due to foreign language, wrong publication type, wrong population, wrong method, or wrong outcome), five publications were full-text screened for inclusion. Finally, three articles were selected for inclusion in the systematic review and meta-analysis. A flow diagram illustrating the selection process is shown in Figure 1.

### 3.3. Study Characteristics and Quality Assessment

This systematic review included three studies published between 2015 and 2020, with a total of 621 HPV-positive male respondents (500 vaccinated and 121 unvaccinated). All three studies were conducted in Italy, with study designs that were unknown (two unreported and one unclear). The largest sample size was in De Toni, 2020 [25], and the smallest in Foresta, 2015 [32] (Table 3). Two studies were rated as good quality, with NOS scores of 8/9 and 7/9 stars, respectively, while the third study was rated as fair quality, with a score of 6/9 stars (Table 3).

The target population was HPV-positive male respondents across all included studies. Their age was reported in two of the three studies (Table 3 and Table 4). The list of evaluated semen parameters was quite the same (total sperm count, progressive motility, and normal morphology in 2/3 studies, sperm concentration, viability, SpermMar test, sperm antibodies, and semen ASA in 1/3 studies each), but they were evaluated for different groups, at different time points, reported in different ways, and expressed in different statistical descriptive measures (Table 1). Sperm concentration and viability were assessed at baseline in only one study for all HPV-positive male respondents, regardless of vaccination status [32]. Total sperm count, progressive motility, and normal morphology were evaluated in two studies at baseline, in one for all HPV-positive male respondents regardless of the vaccination status [32] and in another within: in one for all HPV-positive male respondents regardless of vaccination status [32], and in another for vaccinated respondents only [14]. Garolla et al. also assessed the same semen parameters at three time points (at study initiation, at the end of HPV vaccination, and 6 months after the end of vaccination) [14]. Anti-sperm antibodies (ASA) were measured in all included studies at baseline, for all HPV-positive respondents regardless of vaccination status in one study, and for vaccinated respondents in two studies. Also, results were presented and reported heterogeneously; data regarding semen parameters through time points were presented in figures without numerical reports in the manuscript of Garolla et al., while ASA were expressed as mean and standard error in the same one and as absolute and relative numbers in the remaining two studies [25,32]. ASA were even named differently, as sperm antibodies in Garolla et al. [14], as SpermMar test result in Foresta et al. [32], and as semen ASA in De Toni et al. [25]. Despite previously listed differences, the presence of HPV DNA in semen was evaluated in all included studies using the same method (FISH analysis), reported in the same way (absolute and relative numbers).

### 3.4. Meta-Analysis

Finally, we performed: (1) meta-analysis of HPV DNA negativity in semen in HPV-positive male respondents from baseline to 6 months after the recruitment (at the end of complete vaccination) and from baseline to 12 months after recruitment (6 months after complete vaccination); (2) meta-analysis of the difference in HPV DNA positivity 6 months after recruitment (at the end of complete vaccination), and 12 months after the recruitment (6 months after complete vaccination) between vaccinated and unvaccinated HPV+ male respondents. All respondents received the quadrivalent anti-HPV vaccine as 3 injections over 6 months: the first dose at recruitment, the second 2 months after the first dose, and the third 6 months after the first dose.

**Figure 2:**
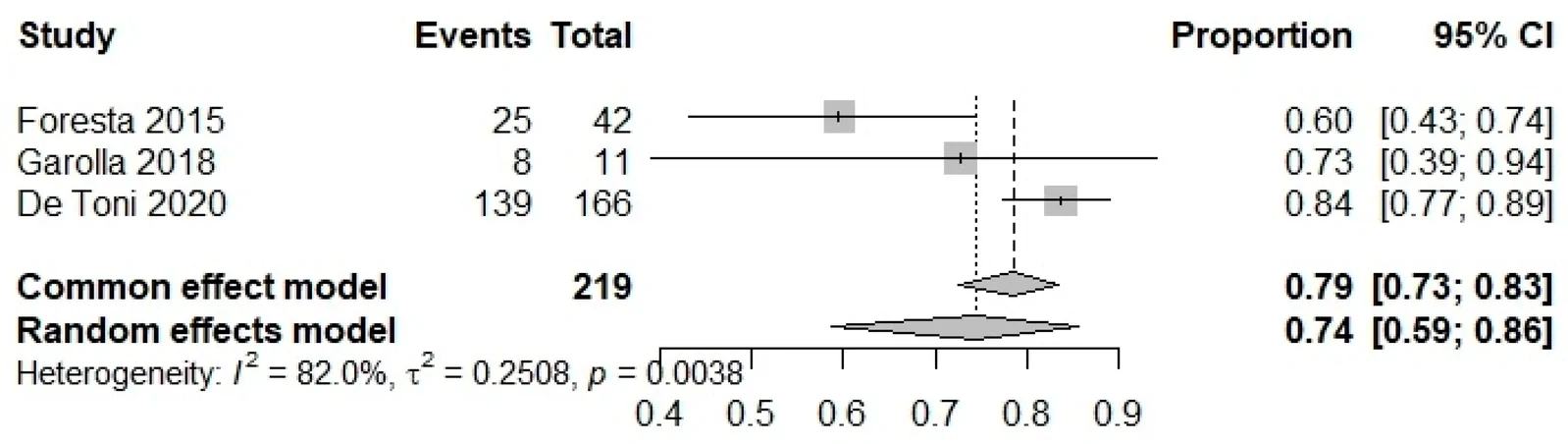
Forest plot of HPV DNA negativity in semen from baseline to 6 months after recruitment (at the end of complete vaccination) [14,25,32].

**Figure 3:**
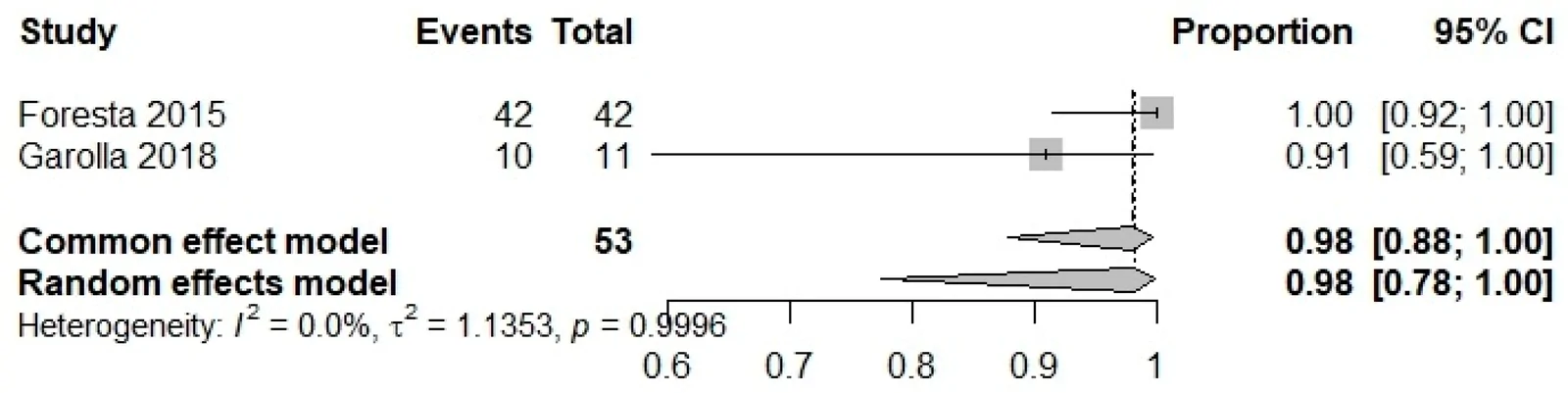
Forest plot of HPV DNA negativity in semen from baseline to 12 months after recruitment (6 months after complete vaccination) [14,32].

Firstly, it was obtained that the estimated overall proportion of HPV DNA negativity was 74% (95% CI 59–86) 6 months after recruitment (at the end of complete vaccination) (Figure 2) and 98% (95%CI 78–100%) 12 months after recruitment (6 months after complete vaccination) (Figure 3).

**Figure 4:**
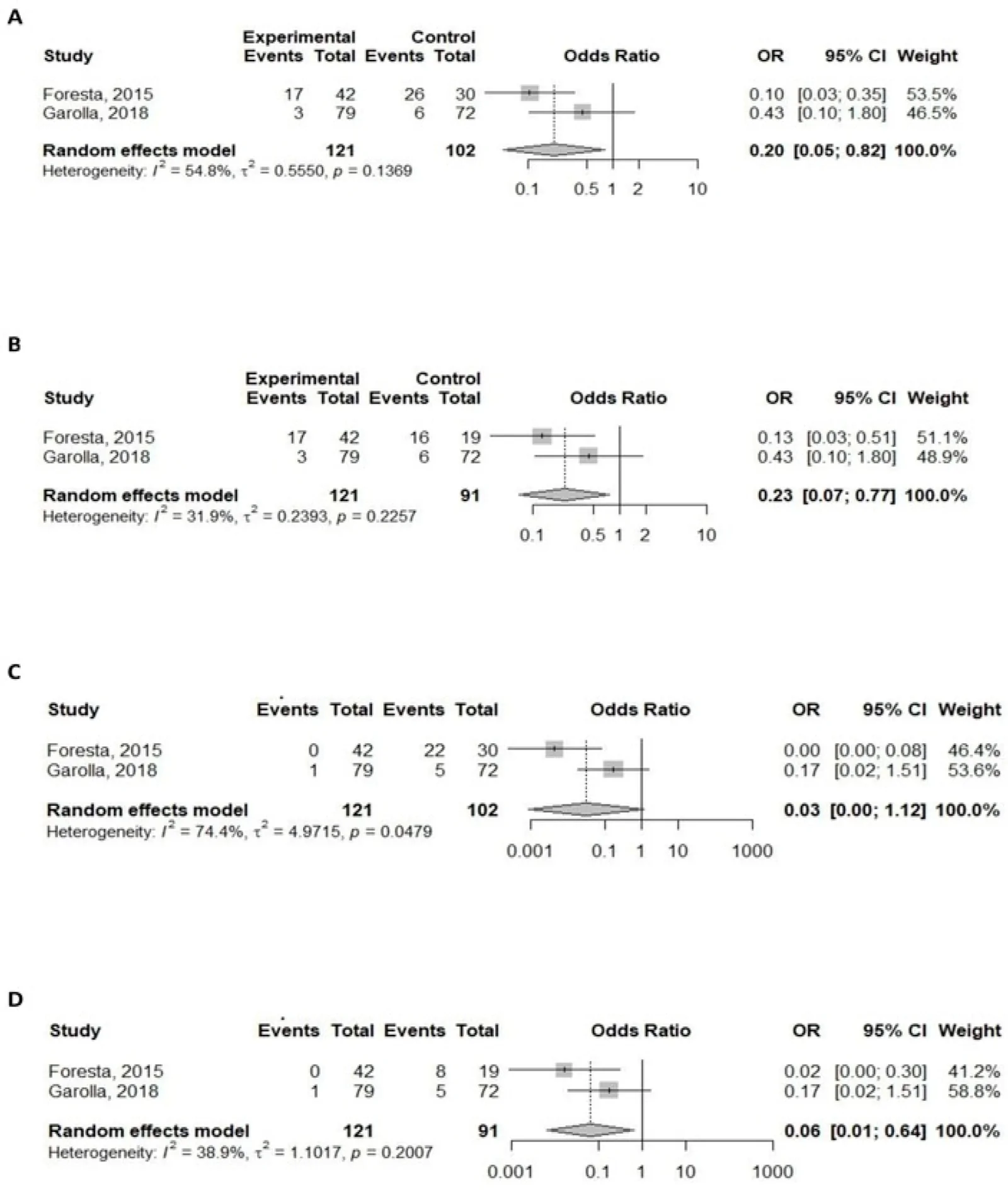
Forest plots of HPV DNA presence in semen among HPV-positive male participants, comparing previously HPV-vaccinated cases with unvaccinated controls: (**A**) 6 months after recruitment, when controls were seronegative in Foresta et al.; (**B**) 6 months after recruitment, when controls were seroconverted in Foresta et al.; (**C**) 12 months after recruitment, when controls were seronegative in Foresta et al.; and (**D**) 12 months after recruitment, when controls were seroconverted in Foresta et al. [14,32].

Secondly, the presence of HPV DNA in semen was significantly lower in vaccinated than in unvaccinated HPV-positive male participants at both 6 and 12 months after recruitment when seroconverted controls from Foresta et al. were considered (Figure 4B,D). When seronegative controls from Foresta et al. were considered, the frequency of HPV DNA-positive samples was significantly lower in vaccinated than in unvaccinated HPV-positive male participants 12 months after recruitment (Figure 4C), whereas no significant difference was observed at 6 months (Figure 4A).

## 4. Discussion

In the absence of an effective and specific anti-HPV therapy, the successful eradication of HPV in patients with persistent infection remains a major clinical challenge. In addition to oncogenic potential, data so far indicate that persistent HPV infection may also be a significant risk factor for impaired fertility outcomes, especially in couples undergoing assisted reproductive techniques [32,35]. Thus, the hypothesis was raised as to whether mitigating fertility impairment associated with persistent HPV infection could be improved if effective HPV clearance were achievable.

Although both viral and host-related factors influence viral persistence, seroconverted patients demonstrate improved HPV clearance compared to seronegative individuals, suggesting that antiviral neutralizing antibodies play a key role in the healing process [32]. Because seroprevalence from natural infection in men is 2.5–3 times lower than in women and is below 20%, the synthesis of anti-HPV-specific antibodies can be more effectively stimulated by vaccination [36,37,38,39]. By stimulating the humoral immune response to the viral L1 protein, the prophylactic HPV vaccine prevents viral infection of basal epithelial cells [40]. Specific neutralizing antibodies recognize the viral surface, offering potential cross-protection against a few non-vaccine HPV types for individuals without previous infection [28]. Initially, it was accepted that the prophylactic HPV vaccine was not effective in treating existing infections or established HPV-related diseases. Moreover, due to the main disparities in vaccination rates between boys and girls, the cost-effectiveness of using the HPV vaccine in males is generally understudied [41].

Studies evaluating the effect of the anti-HPV vaccine on any semen parameter among previously HPV-positive male respondents are scarce. Not even three of the studies included in this systematic review demonstrate homogeneity in the selection of analyzed seminal fluid quality parameters, follow-up intervals to assess the effect of anti-HPV vaccination, or selection of controls. Although all three studies demonstrate that in HPV-positive patients, the quality of seminal fluid is impaired (reduced progressive motility, higher prevalence of positive SperMar test, increased anti-sperm antibodies), due to the heterogeneity of the monitored parameters, a meta-analysis could not be performed [14,25,32]. This could also be supported by Weinberg’s and Cao’s meta-analysis results, which showed a negative effect of HPV on sperm concentration, motility, and morphology [19,20,42]. It should also be noted that the study included in this systematic review, performed by Garolla et al., demonstrated an even higher rate of natural pregnancies and live births in a group of vaccinated male patients [14].

Our two meta-analyses, which evaluated HPV DNA negativity in semen among vaccinated male respondents at two time points, demonstrated a significant reduction in HPV DNA detection following adjuvant administration of a prophylactic HPV vaccine. This reduction was particularly pronounced at the second assessment time point (12 months after recruitment and 6 months after HPV vaccination), at which the pooled estimate shifted toward 98% HPV DNA negativity. An important question arises as to whether the observed indication of viral clearance may be attributable not to a vaccine-induced effect, but rather to the natural history of HPV infection. According to the literature, the time to HPV DNA clearance in unvaccinated individuals is approximately 6 months, and longer for HPV-16 (approximately 12 months) [43,44]. One of the three included studies in our review [25], and the only one without a control group, defined persistent HPV infection for a minimum of six months as an inclusion criterion. Following the authors’ hypothesis, this might be interpreted as a potential failure of effective viral clearance through the natural immune response, thus a potential benefit of vaccination. The other two included studies with control groups were further evaluated and underwent additional meta-analysis comparing HPV DNA positivity between groups. HPV DNA prevalence in semen among vaccinated males was significantly lower compared to unvaccinated HPV-positive male participants at both time points when seroconverted controls were considered, but only after 12 months after recruitment when seronegative controls were considered. Despite these findings being suggestive of supporting the main hypothesis, their interpretation is limited by the lack of information on the control’s serological status before recruitment, including the timing of seroconversion and the duration of HPV infection. Consequently, the potential benefit of vaccination remains difficult to establish in the absence of data on the duration of HPV infection before recruitment of study participants and their vaccination. This may be of particular importance given the known weaker immune response of the keratinized epithelium of the penis than the mucosa of the cervix or anus [45,46]. Men may require a greater number of exposures or to be vaccinated to achieve effective seroconversion [46]. Therefore, a potential role of anti-HPV antibody stimulation in healing from infection may reasonably be hypothesized [32]. A similar effect induced by the HPV vaccine has already been demonstrated in women, reducing the risk of subsequent disease even after surgical treatment [47,48,49,50]. Vaccination effectiveness could be explained by better achievement of the antibody titer threshold value. In particular, one of the studies included in this meta-analysis demonstrated that a serum-antibody titer greater than 1:125 represented a cut-off threshold, identifying patients who achieved complete genital HPV clearance at 6 months [25].

Nevertheless, our analyzed data cannot be considered definitive evidence of HPV DNA clearance but rather evidence of a reduction in detectable HPV DNA, as all included studies used the FISH method for HPV DNA detection. Even previous report has demonstrated a significant correlation between HPV detection by FISH and PCR, FISH has shown lower analytical sensitivity, approximately 84% relative to PCR-positive results [51]. In light of all these considerations, the obtained findings may indicate the potential value of adjuvant administration of a prophylactic HPV vaccine in men with persistent HPV infection within the management of infertility; however, they should be interpreted with caution and regarded as hypothesis-generating rather than confirmatory. Given the small number of available studies and their methodological limitations, further large-scale prospective trials are necessary to establish definitive clinical recommendations.

This study has several limitations. Firstly, there is a lack of studies on the topic of interest, although published original studies as well as narrative reviews indicate its non-negligible importance. Also, for this reason, publication bias could not be reliably assessed. Therefore, the possibility of publication bias cannot be excluded. Secondly, there is heterogeneity mainly arising from study design, including the parameters measured, the timing of their measurement, short duration of follow-up period, inconsistent outcomes and the groups, including controls, in which they were assessed. Given the potential ethical issue of withholding a vaccine that is used for both therapeutic and prophylactic purposes, the challenge of designing studies with vaccinated and unvaccinated groups is evident. However, this could potentially be addressed through a retrospective design (historical cohort), although such an approach is not recommended for conducting high-quality research. In order to help readers quickly grasp the strength of the evidence and avoid overinterpretation, we made a GRADE evidence quality summary for all defined outcomes and presented it in Table 5. Importantly, the certainty of evidence supporting the observed associations was low to very low across the evaluated outcomes, as assessed using the GRADE framework. The certainty of evidence for HPV DNA clearance at 6 and 12 months was rated as very low, primarily due to serious risk of bias and imprecision, reflected by the small number of predominantly observational studies, potential selection bias and confounding, and wide confidence intervals around the pooled estimates. Similarly, the certainty of evidence for the presence of HPV DNA between cases and controls at 12 months was rated as very low, owing to serious risk of bias and imprecision. For the comparison of HPV DNA positivity between cases and controls at 6 months, the certainty of evidence was rated as low, mainly because of the observational nature of the available evidence, potential selection bias and residual confounding, and the limited number of studies. Although the pooled estimates suggested potentially favorable effects associated with vaccination, these findings should therefore be interpreted cautiously, as the available evidence does not allow a high degree of confidence in the estimated effects. Further well-designed, adequately powered prospective studies are needed to confirm these findings and provide more precise estimates of the effect.

## 5. Conclusions

This systematic review evaluated limited evidence suggesting a potential benefit of adjuvant administration of a prophylactic HPV vaccine in infertile HPV-positive men, particularly in terms of shift toward HPV negativity and healing. Considering the scarcity of published data and the methodological limitations of the available studies, despite the strong hypothesis of potential clinical benefit, the current evidence for improvement in semen quality by HPV vaccination remains insufficient and should be critically evaluated. Therefore, large, well-designed prospective trials are required to determine if any clinical management recommendations for this population can be formulated.

## Figures and Tables

**Table 1:** Search strategies.

Database	Search Strategy
**PubMed**	((hpv OR papillomavirus* OR papilloma virus* OR papillomaviridae*) AND (vaccin* OR immunis* OR immuniz* OR Cervarix OR Gardasil OR Valent OR Bivalent OR Quadrivalent OR nine-valent OR 9-valent) AND (male OR man OR men OR male fertility OR infertile men) AND (seminal OR semen OR sperm* OR sperm parameter* OR sperm quality OR motilit* OR morphology* OR concentration* OR count* OR reproductive outcome* OR pregnan* OR live birth*))
**WoS**	((TS=(hpv) OR TS=(papillomavirus*) OR TS=(papilloma virus*) OR TS=(papillomaviridae*)) AND (TS=(vaccin*) OR TS=(immunis*) OR TS=(immuniz*) OR TS=(Cervarix) OR TS=(Gardasil) OR TS=(Valent) OR TS=(Bivalent) OR TS=(Quadrivalent) OR TS=(nine-valent) OR TS=(9-valent)) AND (TS=(male) OR TS=(man) OR TS=(men) OR TS=(male fertility) OR TS=(infertile men)) AND (TS=(seminal) OR TS=(semen) OR TS=(sperm*) OR TS=(sperm parameter*) OR TS=(sperm quality) OR TS=(motilit*) OR TS=(morphology*) OR TS=(concentration*) OR TS=(count*) OR TS=(reproductive outcome*) OR TS=(pregnan*) OR TS=(live birth*)))
**SCOPUS**	((TITLE-ABS-KEY(hpv) OR TITLE-ABS-KEY(papillomavirus*) OR TITLE-ABS-KEY(papilloma virus*) OR TITLE-ABS-KEY(papillomaviridae*)) AND (TITLE-ABS-KEY(vaccin*) OR TITLE-ABS-KEY(immunis*) OR TITLE-ABS-KEY(immuniz*) OR TITLE-ABS-KEY(Cervarix) OR TITLE-ABS-KEY(Gardasil) OR TITLE-ABS-KEY(Valent) OR TITLE-ABS-KEY(Bivalent) OR TITLE-ABS-KEY(Quadrivalent) OR TITLE-ABS-KEY(nine-valent) OR TITLE-ABS-KEY(9-valent)) AND (TITLE-ABS-KEY(male) OR TITLE-ABS-KEY(man) OR TITLE-ABS-KEY(men) OR TITLE-ABS-KEY(male fertility) OR TITLE-ABS-KEY(infertile men)) AND (TITLE-ABS-KEY(seminal) OR TITLE-ABS-KEY(semen) OR TITLE-ABS-KEY(sperm*) OR TITLE-ABS-KEY(sperm parameter*) OR TITLE-ABS-KEY(sperm quality) OR TITLE-ABS-KEY(motilit*) OR TITLE-ABS-KEY(morphology*) OR TITLE-ABS-KEY(concentration*) OR TITLE-ABS-KEY(count*) OR TITLE-ABS-KEY(reproductive outcome*) OR TITLE-ABS-KEY(pregnan*) OR TITLE-ABS-KEY(live birth*)))

* Truncation using an asterisk (*) was applied to the word root to identify terms with different prefixes and/or suffixes.

**Table 2:** Feasibility assessment of meta-analysis.

Component	
Research Question and Objectives	The research question was defined as follows: Does any anti-HPV vaccine improve any of the semen characteristics among HPV-positive male respondents? Objectives: Primary—To evaluate the difference in count, frequency, or level of semen characteristics before and after anti-HPV vaccination. Secondary—To evaluate the difference in count, frequency, or level of semen characteristics between vaccinated and unvaccinated respondents.
Availability of Data	Search 3 electronic databases: PubMed, SCOPUS, and Web of Science A total of 2957 articles were collected. After duplicates were removed, titles and abstracts were screened for 1802 articles. Inclusion criteria: Population—Male human HPV-positive respondents of all ages Intervention—anti-HPV vaccine Control—placebo or no intervention Outcome—sperm concentration, total sperm count, progressive motility, normal morphology, viability, IgG or IgM antibodies, and HPV in semen Study design—clinical trials, prospective or retrospective cohorts, nested case–controls in cohorts, case–controls, and cross-sectionals Exclusion criteria: Language other than English Non-original articles (narrative reviews, systematic reviews, meta-analyses, case reports, case series, editorials, comments, correspondence, books, short abstracts, etc.) Population other than humans (animals, cell lines) Other vaccine than anti-HPV semen characteristics of interest were not evaluated at all 3 articles were included in qualitative synthesis.
Homogeneity of Studies	Studies were completely homogeneous regarding the type and number of received doses of prophylactic quadrivalent anti-HPV vaccine, as well as vaccine manufacturer (Gardasil (Merck Serono S.p.A., Milan, Italy) Moderate heterogeneity was present regarding: - study design (1 design was unclear, and 2 did not report their design) - study population (infertile male respondents in 2, and just male respondents in 1 study) - inclusion of HPV genotypes covered by cross-protection (2 studies included and 1 did not) - design of examining semen parameters as outcomes (1 evaluated for all respondents at baseline, 1 in vaccinated and non-vaccinated and during follow-up period, and 1 for all during the follow-up period)
Methodological Quality	Was evaluated by the Newcastle–Ottawa scale system for observational studies and the Jadad scale for clinical trials. All 3 included studies had low quality.
Heterogeneity Assessment	Major findings were not so heterogeneous regarding the presence of HPV DNA in semen; thus, a meta-analysis was possible for this outcome.
Statistical Feasibility	There were different ways of testing differences between groups or time points during the follow-up period. Statistical tests were applied; thus, *p*-values were reported.
Publication Bias	Was assessed by funnel plot. It was absent.
Resource Availability	Technical resources (3 electronic databases) and human resources (2 independent reviewers, with a third for resolving disagreements) were available without restrictions.
Ethical Considerations	We can think about ethical questions regarding not giving an anti-HPV vaccine if it is recommended in the target population. That could explain the choice of a cohort study design rather than a case–control design, which is certainly the more appropriate choice in this context.
Practicality of Results	The results of the meta-analysis are expected to demonstrate the therapeutic efficacy of anti-HPV vaccination in infertile HPV-positive men in terms of improvements in semen parameters.

**Table 3:** Systematic review table—study characteristics with outcomes.

Author, Year Country Study Design NOS Score	n Cases vs. Controls Age Cases vs. Controls	Semen Characteristic (Unit)	Values of Semen Parameters (Time of the Analysis)		Semen HPV DNA—FISH Analysis (Time of the Analysis)	
			Cases	Controls	Cases	Controls
Foresta, 2015 [32] Italy Not reported 8/9	42 vs. 49 (19 seroconverted + 30 seronegative) NR vs. NR	Sperm concentration (×10^6^/^mL^)	30.4 ± 13.1 (at baseline)	Seroconverted 29.9 ± 7.9 (at baseline) Seronegative 33.4 ± 15.2 (at baseline)	At recruitment 42/42 3 months after recruitment 31/42 6 months after recruitment 17/42 9 months after recruitment 3/42 12 months after recruitment 0/42 18 months after recruitment 0/42 24 months after recruitment 0/42	Seroconverted At recruitment 19/19 3 months after recruitment Not performed 6 months after recruitment 16/19 9 months after recruitment Not performed 12 months after recruitment 8/19 18 months after recruitment 1/19 24 months after recruitment 0/19 Seronegative At recruitment 30/30 3 months after recruitment Not performed 6 months after recruitment 26/30 9 months after recruitment Not performed 12 months after recruitment 22/30 18 months after recruitment 10/30 24 months after recruitment 7/30
		Total sperm count (×10^6^)	92.9 ± 40.3 (at baseline)	Seroconverted 90.7 ± 42.6 (at baseline) Seronegative 93.4 ± 39.8 (at baseline)		
		Progressive motility (%)	22.7 ± 13.4 (at baseline)	Seroconverted 15.4 ± 9.5 (at baseline) Seronegative 23.1 ± 14.2 (at baseline)		
		Normal morphology (%)	14.9 ± 8.7 (at baseline)	Seroconverted 14.8 ± 10.0 (at baseline) Seronegative 15.1 ± 8.9 (at baseline)		
		Viability (%)	74.9 ± 9.8 (at baseline)	Seroconverted 75.2 ± 7.7 (at baseline) Seronegative 74.4 ± 10.1 (at baseline)		
		Positive SpermMar test (%)	88 (49.2) (at baseline)	Seroconverted 18 (94.7%) (at baseline) Seronegative 70 (43.8%) (at baseline)		
Garolla, 2018 [14] Italy Not clear (retrospective study) 7/9	79 vs. 72 32.4 ± 3.1 vs. 32.8 ± 2.8	Total sperm count (×10^6^/^mL^)	At baseline 165.1 ± 163.9 At the end of HPV vaccination (6 months from baseline) 146.3 ± 203.9 6 months after HPV vaccination (12 months from baseline) 152.8 ± 152.9	At baseline 182.6 ± 128.1 At the end of HPV vaccination (6 months from baseline) 170.4 ± 165.5 6 months after HPV vaccination (12 months from baseline) 173.5 ± 204.5	At baseline 11/79 At the end of HPV vaccination (6 months from baseline) 3/79 6 months after HPV vaccination (12 months from baseline) 1/79	At baseline 10/72 At the end of HPV vaccination (6 months from baseline) 6/72 6 months after HPV vaccination (12 months from baseline) 5/72
		Progressive motility (%)	At baseline 31.6 ± 15.5 At the end of HPV vaccination (6 months from baseline) 40.7 ± 28.9 6 months after HPV vaccination (12 months from baseline) 43.5 ± 26.7	At baseline 32.3 ± 15.1 At the end of HPV vaccination (6 months from baseline) 32.0 ± 26.7 6 months after HPV vaccination (12 months from baseline) 31.8 ± 28.4		
		Normal morphology (%)	At baseline 10.2 ± 6.9 At the end of HPV vaccination (6 months from baseline) 9.7 ± 12.4 6 months after HPV vaccination (12 months from baseline) 11.0 ± 14.2	At baseline 11.7 ± 5.8 At the end of HPV vaccination (6 months from baseline) 10.5 ± 13.6 6 months after HPV vaccination (12 months from baseline) 10.9 ± 14.4		
		Sperm antibodies (%)	At baseline 9.8 ± 20.3 At the end of HPV vaccination (6 months from baseline) 4.0 ± 21.3 6 months after HPV vaccination (12 months from baseline) 3.7 ± 16.9	At baseline 12.4 ± 23.4 At the end of HPV vaccination (6 months from baseline) 11.6 ± 21.2 6 months after HPV vaccination (12 months from baseline) 10.8 ± 26.7		
De Toni, 2020 [25] Italy Not reported 6/9	379 vs. No control group 40.3 ± 0.65 vs. No control group	Semen ASA (%)	At baseline 88/379 (23.2%) 6 months after HPV vaccination 54/379 (14.2%)	No control group	At baseline 166/379 6 months after HPV vaccination 27/379	No control group

**Table 4:** Respondents’ characteristics.

Study	Cases			Controls
	Characteristics	Criteria for Inclusion	Criteria for Exclusion	Characteristics
Foresta, 2015 [32]	Male patients attending the unit for assisted reproduction who were vaccine sensitive (according to cross-protection provided by quadrivalent vaccine patients featured by infection of at least one of the HPV types 6, 11, 16, 18, 31, 33, 45, 52, and 58)	Diagnosis of male infertility was defined as alteration of sperm parameters and/or at least 2 years of unprotected sexual intercourse without conception with female partners without major genital diseases (tubal factor, uterine factor, cervical abnormalities, and ovarian disorders were excluded)	History of cryptorchidism, testicular trauma, post-mumps orchitis, prior knowledge of HPV infection, previous or ongoing vaccination at the time of enrolment, varicocele and bacterial seminal infections	Seroconverted for vaccine-type HPV at recruitment. Seronegative vaccine-sensitive patients at recruitment
Garolla, 2018 [14]	Male partners aged 25 to 40 years	HPV semen infection and total sperm count ≥40 × 10^6^ cells per ejaculate, independently of the other sperm parameters evaluated according to World Health Organization guidelines 2010	Concomitant infection of *Chlamydia trachomatis*, *Ureaplasma urealyticum*, *Neisseria gonorrhoeae* or other sperm infections, seropositivity toward human immunodeficiency virus type 1 or 2, human T-cell lymphotropic virus type 1 or 2, hepatitis B or C virus and *Treponema pallidum*, karyotype abnormalities or CFTR mutations	Non-vaccinated patients
De Toni, 2020 [25]	Males attending the unit for andrological issues (genital warts and/or infertility associated with risk factors for HPV infection such as previous warts, HPV infected partner, idiopathic asthenozoospermia, anti-sperm antibodies)	Acceptance of written informed consent for clinical data access, persistent HPV infection (at least 6 months) from genotypes included in the quadrivalent vaccine, such us 6, 11, 16, 18, or those with evidence of cross-protection, such as 31, 33, 45, 52 and 58 and patients receiving HPV vaccination (3 doses of, quadrivalent vaccine, administered respectively at study initiation, after 2 months and after 6 months from first dose)	Presence of any other concomitant sexually transmitted infections of the semen (*Chlamydia trachomatis*, *Neisseria gonorrhoeae*, *Mycoplasma* spp.), Human Immunodeficiency Virus (HIV), Hepatitis B Virus (HBV), Hepatitis C Virus (HCV) and *Treponema pallidum* active infections, CFTR gene mutations and any alteration of the major endocrine axes	No control group

**Table 5:** GRADE evidence quality summary.

Outcome	Main Risk of Bias	Inconsistency	Indirectness	Imprecision	Publication Bias	Limitations	GRADE Certainty of Evidence
**HPV DNA clearance 6 months after recruitment (at the end of complete vaccination)**	Serious	Not serious	Not serious	Serious	NA	Small number of studies; predominantly observational evidence; potential selection bias and confounding; relatively small sample sizes; wide CI around the pooled estimate	**Very low**
**HPV DNA clearance after 12 months (6 months after complete vaccination)**	Serious	Not serious	Not serious	Serious	NA	Only two studies; observational evidence; potential selection bias and confounding; very wide CI despite a high pooled clearance estimate	**Very low**
**Presence of HPV DNA between cases and controls 6 months after recruitment (at the end of complete vaccination)**	Serious	Not serious	Not serious	Not serious	NA	Only two observational studies; potential selection bias and residual confounding; limited evidence base	**Low**
**Presence of HPV DNA between cases and controls 12 months after recruitment (6 months after complete vaccination)**	Serious	Not serious	Not serious	Serious	NA	Only two observational studies; potential selection bias and residual confounding; relatively small evidence base; wide CI around the pooled OR	**Very low**

Abbreviation: NA—not applicable.

## Data Availability

The data presented in this study are available on request from the corresponding author.

## Abbreviations

The following abbreviations are used in this manuscript:

HPV	Human papillomavirus
DNA	Deoxyribonucleic acid
ASA	Anti-sperm antibodies
NR	Not reported
FISH	Fluorescence In Situ Hybridization
